# Copper Nanoparticle and Nitrogen Doped Graphite Oxide Based Biosensor for the Sensitive Determination of Glucose

**DOI:** 10.3390/nano8060429

**Published:** 2018-06-13

**Authors:** Kulandaivel Sivasankar, Karuppasamy Kohila Rani, Sea-Fue Wang, Rajkumar Devasenathipathy, Chia-Her Lin

**Affiliations:** 1 Department of Chemistry, Chung-Yuan Christian University, Chungli District, Taoyuan City 32023, Taiwan; sivasankarmpm@gmail.com; 2 Department of Materials and Mineral Resources Engineering, National Taipei University of Technology, No. 1, Sec. 3, Chung-Hsiao East Rd., Taipei 106, Taiwan; kokirackz@gmail.com (K.K.R.); chemrackz@gmail.com (R.D.); 3 R&D Center for Membrane Technology, Chung-Yuan Christian University, Chungli District, Taoyuan City 32023, Taiwan

**Keywords:** metal-organic framework, copper nanoparticles, nitrogen doped graphite oxide, amperometric techniques, glucose, human serum samples

## Abstract

Copper nanoparticles with the diameter of 50 ± 20 nm decorated nitrogen doped graphite oxide (NGO) have been prepared through a simple single step carbonization method using copper metal-organic framework (MOF), [Cu_2_(BDC)_2_(DABCO)] (where BDC is 1,4-benzenedicarboxylate, and DABCO is 1,4-Diazabicyclo[2.2.2]octane) as precursor. The surface morphology, porosity, surface area and elemental composition of CuNPs/NGO were characterized by various techniques. The as-synthesized CuNPs/NGO nanomaterials were coated on commercially available disposable screen-printed carbon electrode for the sensitive determination of glucose. We find that the modified electrode can detect glucose between 1 μM and 1803 μM (linear range) with good sensitivity (2500 μA mM^−1^ cm^−2^). Our glucose sensor also possesses low limits of detection (0.44 μM) towards glucose determination. The highly selective nature of the fabricated electrode was clearly visible from the selectivity studies. The practicability of CuNPs/NGO modified electrode has been validated in the human serum samples. The storage stability along with better repeatability and reproducibility results additionally substantiate the superior electrocatalytic activity of our constructed sensor towards glucose.

## 1. Introduction

At present, the development of glucose biosensors have received more interest in the diagnosis of human blood sugar level, food industry and waste water treatment [1,2]. The detection of glucose has been performed using several methods such as fluorescent spectrometer [3], liquid chromatography [4], and electrochemical sensors [5]. Compared to other methods, electrochemical methods have gained the preference in bio-sensing research because of their facile approach, good repeatability, high selectivity and low cost [6]. The miniscule screen printed carbon electrodes (SPCEs) have been introduced in the development of fast and accurate sensing devices. These SPCEs are non-reusable, cost effective and are also comprised of three electrodes on planar strips [7,8,9]. The electrochemical experiments involving SPCEs have several advantages such as: (i) no effect of oxygen interference (ii) measurements in micro-volumes of sample solutions are possible and (iii) no need for mechanical polishing of electrode surface [5]. In contrast to these advantages, the use of bare (unmodified) electrodes in the enzyme less electrochemical detection leads to certain drawbacks like poor electron transfer, high over potential and electrode fouling. The surface of SPCEs has been chemically modified by the present-day researchers to sense the desirable analytes [7]. These modified electrodes are chosen to overcome the above shortcomings in the non-enzymatic electrochemical determination of glucose [10,11].

Several carbon based metal and metal oxide have been utilized for the fabrication of non-enzymatic glucose biosensors as they possess high surface area, good sensitivity, selectivity and electrocatalytic activity towards glucose. Advantages such as non-toxicity and low cost of copper have attracted the researchers in the fabrication of low cost biosensors [12,13] compared to noble metals (Au, Pt and Pd) [14,15,16]. Notably, these copper based nanomaterials show higher catalytic activity towards the oxidation of glucose than that of other common metal nanomaterials such as nickel, manganese, cobalt, etc. [17,18,19]. Therefore, many synthetic protocols have been developed for the preparation of copper based nanocomposites. In order to increase the dispersion and decrease the aggregation of copper nanoparticles, certain carbon materials (graphene, carbon nanotubes (CNTs), and carbon block, etc.) are used as matrices by the current researchers [12,20,21]. Interestingly, N-doped carbon materials provide high surface area and a large number of active sites for the incorporation of copper nanoparticles without any aggregation. For example, Ding et al. synthesized copper nanoparticles decorated N-doped graphene for the fabrication of glucose biosensor where they reported an enhanced electrochemical activity towards glucose compared only copper nanoparticles [22]. However, the preparation of electrodes based on binder-free carbon/Cu composite materials remains challenging.

Metal-organic frameworks (MOFs) are one of the industrialized hybrid porous materials and it has a buildup of metal ions/clusters linked through organic linkers, which presents a diverse network architecture, topology, desirable pore size and high surface area. Along with many application perceptive, MOFs are utilized in heterogeneous catalysis [23], gas storage [24], colorimetric bio sensing [25] and drug delivery [26]. In particular, MOFs are attractive candidates for sensing and biosensing applications, for example: electrocatalyst supporting matrix for electrochemical sensors in the detection of glucose, acetaminophen dopamine, nicotinamide adenine dinucleotide (NADH), catechol and hydroquinone hydrogen peroxide, and cysteine [27,28,29,30,31,32,33,34]. However, most of the MOF are unstable in air moisture, and, hence, their water-based application has been limited. In order to overcome the above issue, MOFs have been carbonized under inert atmosphere to produce the metal- or metal oxide-embedded carbon composite materials, named carbonized MOFs (CMOFs). Furthermore, the CMOFs showed high surface areas and ordered pores as like their parent MOFs, which is well-suited for many applications including biosensors.

Motivated by the benefits of CMOFs, this work presents the synthesis of copper nanoparticles decorated with nitrogen doped graphite oxide (CuNPs/NGO) (without addition of binder) through direct carbonization of a copper MOF, [Cu_2_(BDC)_2_(DABCO)] (where BDC is 1,4-benzenedicarboxylate, and DABCO is 1,4-diazabicyclo[2.2.2]octane). Various carbonization temperatures (700, 800, and 900 °C) were used to study the effect of temperature in the synthesis of CuNPs/NGO. The prepared CuNPs/NGO were coated on screen-printed carbon electrode for the fabrication of electrochemical sensors. Our fabricated CuNPs/NGO/SPCE showed a good electrochemical activity of CuNPs/NGO towards the determination of glucose. The obtained electroanalytical performances in terms of limits of detection (LOD), quick amperometric response, high sensitivity, attractive selectivity and a wide linear range are good or comparable with formerly reported literature Moreover, the accurate determination of glucose in human serum samples confirms the practical feasibility of CuNPs/NGO nanocomposite.

## 2. Experimental Section

### 2.1. Materials and Methods

Copper (III) nitrate (Cu(NO_3_)_2_·3H_2_O, Showa (≥99.9%), Tokyo, Japan), dimethylformamide (DMF, Merck (≥99.8%), Darmstdt, Germany), 1,4-benzene dicarboxylate (BDC, Sigma-Aldrich (98%), Burlington, MA, USA), 1,4-diazabicyclo[2.2.2]octane (DABCO, Alfa Aesar (98%), Lancashire, UK), glucose and sodium hydroxide (NaOH) were purchased from Sigma-Aldrich (Darmstdt, Germany) at an analytical grade. The electrochemical experiments were done using 0.1 M NaOH as the supporting electrolyte. Prior to each experiment, all the solutions were deoxygenated with pre-purified N_2_ gas for 15 min. All of the electrochemical measurements were carried out with double distilled water, which has a conductivity of ≥18 MΩ cm. Human blood serum sample was collected from valley biomedical, Taiwan product & services, Inc. This study was reviewed and approved by the ethics committee of Chang-Gung memorial hospital through the contract no. IRB101-5042A3.

### 2.2. Apparatus

The phase purity of all the compounds was examined by powder X-ray diffraction (PXRD) using a Bruker D8 PHASER instrument (Billerica, MA, USA). The synthesized CMOFs were also characterized using a micro-Raman module with a He–Ne laser (632.8 nm). The charge-coupled device (CCD) exposure time was varied from 5 to 20 s. Raman shifts were calibrated using the silicon (Si) reference peak at 521 cm^−1^. The N_2_ gas sorption isotherms were measured at 77 K using an ASAP 2020 system of Micrometrics (Norcross, GA, USA). Ultrahigh purity grade N_2_, and He were used as received. Before the gas sorption measurements, the sample was initially dehydrated at 423 K for 24 h under vacuum. A high resolution scanning electron microscopy (HRSEM, using a JEOL JEM-7600F instrument, Akishima, Japan) and transmission electron microscopy (TEM, using a JEM-2010 instrument, Tokyo, Japan) were employed to characterize the morphology. The Cu content was analyzed by using an Inductively Coupled Plasma-Mass Spectrometer (ICP-MS) (Japan Agilent 7500ce, Tokyo, Japan) after the sample was dissolved, and the elemental analysis was performed on an Elementar vario EL III CHN-OS elemental analyzer (Germany). The electrochemical measurements were carried out through CHI 6171D work station with a conventional three electrode cell, which uses modified SPCE (area = 0.071 cm^2^), saturated Ag|AgCl (saturated KCl) and Pt wire as working, reference and counter electrodes, respectively. An analytical rotator AFMSRX (PINE instruments, Grove City, PA, USA) with a rotating disc glassy carbon electrode (RDE, area = 0.21 cm^2^) was utilized in the amperometric i–t measurements.

### 2.3. Preparation of [Cu_2_(BDC)_2_(DABCO)]

All reagents and solvents employed were used without any further purification. The bulk MOF, [Cu_2_(BDC)_2_(DABCO)] materials were synthesized by solvothermal methods as described in literature [35]. Typically, a mixture of Cu(NO_3_)_2_·3H_2_O (3 mmol, 725 mg), H_2_BDC (3 mmol, 498 mg), and DABCO (2.49 mmol, 279 mg) was taken in a conical flask containing 60 mL of DMF and stirred at room temperature for 20 min, followed by 20 min of sonication. The mixture was transferred into a Teflon-lined autoclave and heated at 120 °C for 48 h. After that, the mixture was cooled to room temperature. The blue colored solid product was filtered, washed thoroughly with DMF for the removal of unreacted reagents and dried overnight at room temperature under vacuum. The dried material was transferred into a vacuum desiccator and further used for the preparation of carbonized MOF (CMOF).

### 2.4. Preparation of CuNPs/NGO

The CuNPs/NGO materials were synthesized through simple one-step direct carbonization method. In addition, 400 mg of [Cu_2_(BDC)_2_(DABCO)] was carefully taken in a silica boat and placed inside the furnace chamber. The chamber was evacuated and N_2_ gas was passed in to the chamber for 1 h to create N_2_ atmosphere. Then, the MOF was heated to 600 °C under N_2_ atmosphere at a heating rate of 5 °C min^−1^. The temperature was maintained at 600 °C for 5 h and then cooled to room temperature with a cooling rate of 1 °C min^−1^. The carbonization of [Cu_2_(BDC)_2_(DABCO)] MOF under N_2_ atmosphere leads to the formation of copper nanoparticles decorated with Nitrogen- functionalized graphite oxide (CuNPs/NGO). The resulting carbonized sample was transferred to a sample tube, sealed with paraffin film and noted as CuNPs/NGO (600) based on the carbonization temperature. By following the similar procedure, CuNPs/NGO (700), CuNPs/NGO (800) and CuNPs/NGO (900) were also prepared.

### 2.5. Electrode Fabrication

Furthermore, 2 mg of all the carbonized CuNPs/NGO samples was individually dispersed in 1 mL of DMF. The electrode (glassy carbon electrode) surface was pre-polished with a Buehler polishing kit (MicroCloth, Magnetic, 8 in, Tokyo, Japan) using ET033 0.05 µm alumina slurry, washed and air-dried in the oven. Later, the 10 μL of CuNPs/NGO sample was drop cast onto the surface SPCE and dried at suitable conditions. The resulting modified electrode was utilized for the electrochemical studies.

## 3. Results and Discussion

### 3.1. Formation of [Cu_2_(BDC)_2_(DABCO)]

**Figure 1:**
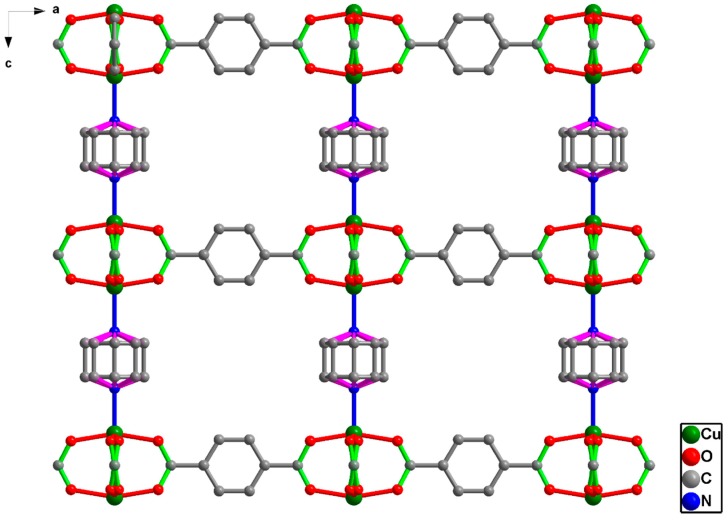
Crystal structure of [Cu_2_(BDC)_2_(DABCO)] along the *b*-axis (hydrogen atoms are not given for the sake of clarity).

The precursor MOF, [Cu_2_(BDC)_2_(DABCO)] for the construction of Cu nanoparticle embedded nitrogen functionalized graphite oxide materials is comprised of dinuclear Cu_2_ units with a paddle wheel structure, bridged by BDC dianions forms a distorted 2D square-grid [Cu_2_(BDC)_2_]. The axial sites of Cu_2_ paddle wheels are occupied by DABCO, which acts as pillars for the extension of 2D layers into a 3D structure (Figure 1). Accordingly, [Cu_2_(BDC)_2_(DABCO)] MOF was prudently selected as the nitrogen functionalized graphite oxide precursor. [Cu_2_(BDC)_2_(DABCO)] prepared under the solvothermal conditions exhibits a pure phase of crystalline material, as-synthesized PXRD patterns showed in Figure S1 and N_2_ gas sorption technique was used to determine the surface area of prepared MOF [Cu_2_(BDC)_2_(DABCO)] in Figure S2.

### 3.2. Characterization of Prepared CuNPs/NGO Nanocomposite

**Figure 2:**
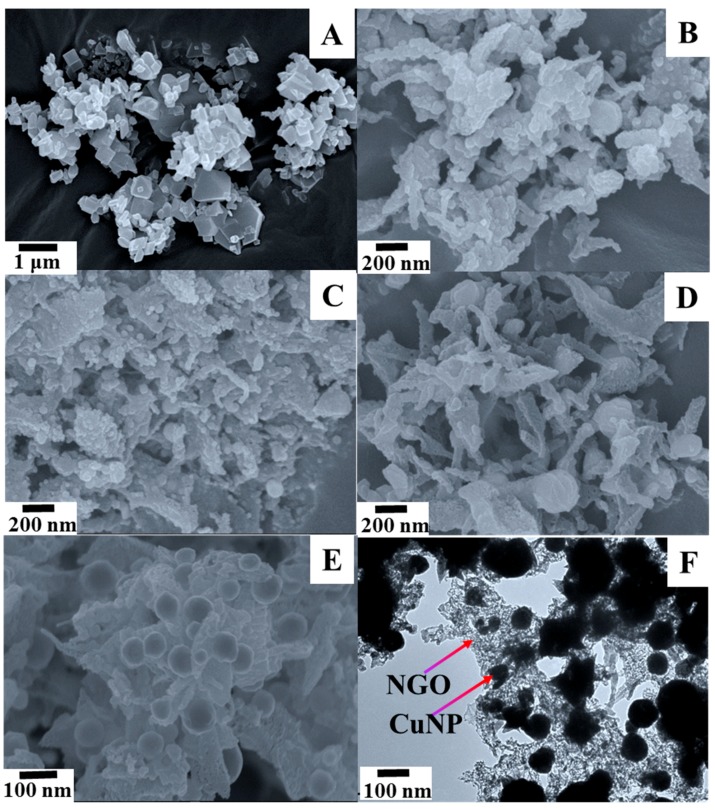
SEM images of only MOF (**A**); CuNPs/NGO (carbonized at 600 °C (**B**); 700 °C (**C**); 800 °C (**D**); 900 °C (**E**); and TEM image (**F**) of CuNPs/NGO (900 °C)).

The morphologies of Cu nanoparticles decorated nitrogen doped graphite oxide were studied by scanning electron microscopy (SEM) and transmission electron microscopy (TEM). The SEM image of the prepared parent MOF [Cu_2_(BDC)_2_(DABCO)] displayed the single uniform trigonal and tetragonal structures (Figure 2A), whereas the SEM images of CuNPs/NGO (carbonized at 600 °C (B), 700 °C (C), 800 °C (D), 900 °C (E)) clearly illustrate that the composite also comprises Cu and Cu_2_O nanoparticles along with the amorphous carbon bed. In the composite material carbonized at high temperature (900 °C), Cu nanoparticles are non-uniformly (diameter of 50 ± 20 nm) embedded into the amorphous carbon bed. This was supported by the TEM image in Figure 2F.

The combined study of inductively coupled Plasma-Mass spectrometry (ICP-MS) and Elemental analysis (EA) reveals the weight percentages of various elements present in CuNPs/NGO composite (Table 1). Detailed elemental information about copper, nitrogen, carbon and oxygen confirms the high weight percentage of copper relative to other elements. The lower weight percentage of oxygen compared to copper supports the formation of copper nanoparticles instead of Cu_2_O. The occurrence of nitrogen in appreciable quantity indicates the nitrogen functionalization in the prepared composite. This available nitrogen content can enhance the electrical conductivity of CuNPs/NGO and the nitrogen containing functional groups can probably react with glucose molecules through van der Waals forces as well as hydrogen bonding.

**Figure 3:**
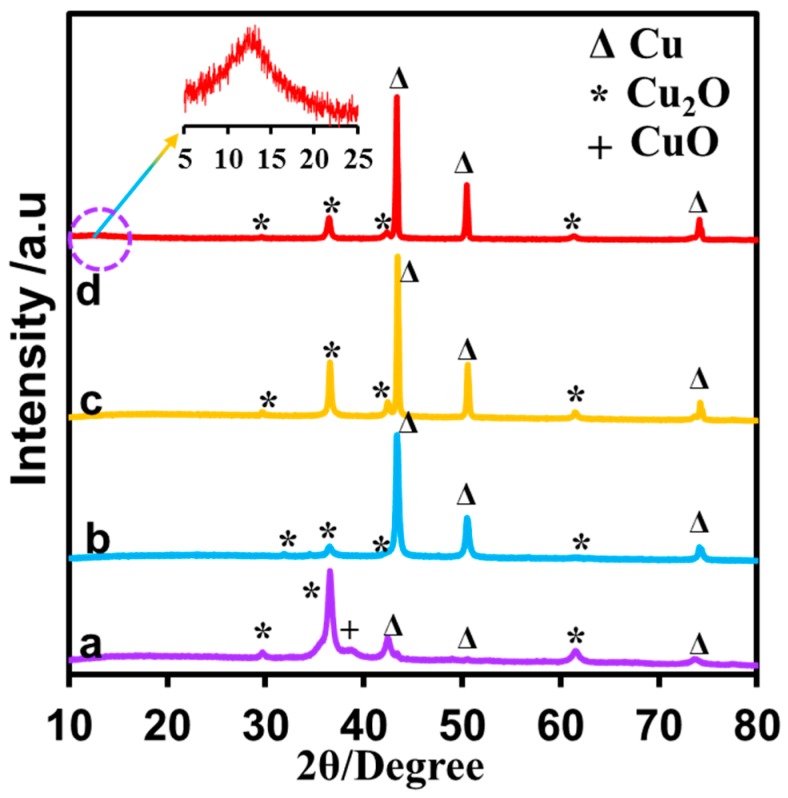
XRD pattern of CuNPs/NGO (carbonized at 600 °C (**a**), 700 °C (**b**), 800 °C (**c**) and 900 °C (**d**)).

The crystal structure and the phase purity of the carbonized products were examined through Powder X-ray diffraction (PXRD) and presented in Figure 3. The obtained PXRD patterns were consistent with the pattern of CuNPs/NGO composite. The enhanced diffraction peaks at 43.33°, 50.47° and 74.12° can be assigned to the (111), (200) and (220) crystal planes of Cu ^(0)^ (Joint Committee on Powder Diffraction Standards—JCPDS) (JCPDS, No. 65-9026) [36]. The diffraction peaks at 29.63°, 36.43°, 42.33° and 61.52° can be assigned to the (110), (111), (200) and (220) planes of Cu_2_O (JCPDS, No. 05-0667). The particle size of Cu_2_O particles estimated from the Scherer equation using the PXRD data was approximately 12 nm at 600 °C, and increased to 26, 44 and 50 nm at 700, 800 and 900 °C, respectively. Notably, the formation of Cu_2_O is higher in CuNPs/NGO (600), but the formation of Cu ^(0)^ is higher in CuNPs/NGO (900). It indicates that the copper reduction (from Cu ^(II)^–>Cu ^(0)^) was increased upon increasing of annealing temperature from 600 °C to 900 °C. Moreover, a new peak at 11.8° was also observed in the CuNPs/NGO(900), which can be due to the (001) reflection of graphite oxide (GO) [37].

The low degree graphitization of nitrogen-functionalized graphite oxide material was further characterized by Raman spectroscopy. The D and G bands between 1200 cm^−1^ and 1600 cm^−1^ were analyzed by fitting with Gaussian profiles. Gaussian fitting possesses better fitting accuracy and was also used in analyzing the presence of amorphous carbons. Thus, the simultaneous fitting of the spectra with four bands has been accomplished: 1180 cm^−1^ (A1 band), 1350 cm^−1^ (D band), 1500 cm^−1^ (A2 band), and 1580 cm^−1^ (G band). The Raman spectrum in CuNPs/NGO (carbonized at 600 °C (a), 700 °C (b), 800 °C (c) and 900 °C (d)) represents the combination of A1 and D bands as a broad peak and so the A1 and D band separation as well as the determination of D and G peak positions were achieved using Gaussian fitting after the baseline subtraction (Figure S3). D band intensity shows the existence of defects, edge effects and dangling sp^2^ carbon bonds that break the symmetry and disorder-induced effects for any type of carbon. The appearance of the *G* peak is due to the in-plane stretching motion between sp^2^ carbon atoms and *A*_2_ band, an indication of the existence of amorphous carbon [38]. The order of intensity ratio (*I*_D_/*I*_G_ ratio) is CuNPs/NGO (600) < CuNPs/NGO (700) < CuNPs/NGO (800) < CuNPs/NGO (900). This indicates the formation of disorderliness, defect carbons and low degree of graphitization in the obtained CuNPs/NGO (900) material.

**Figure 4:**
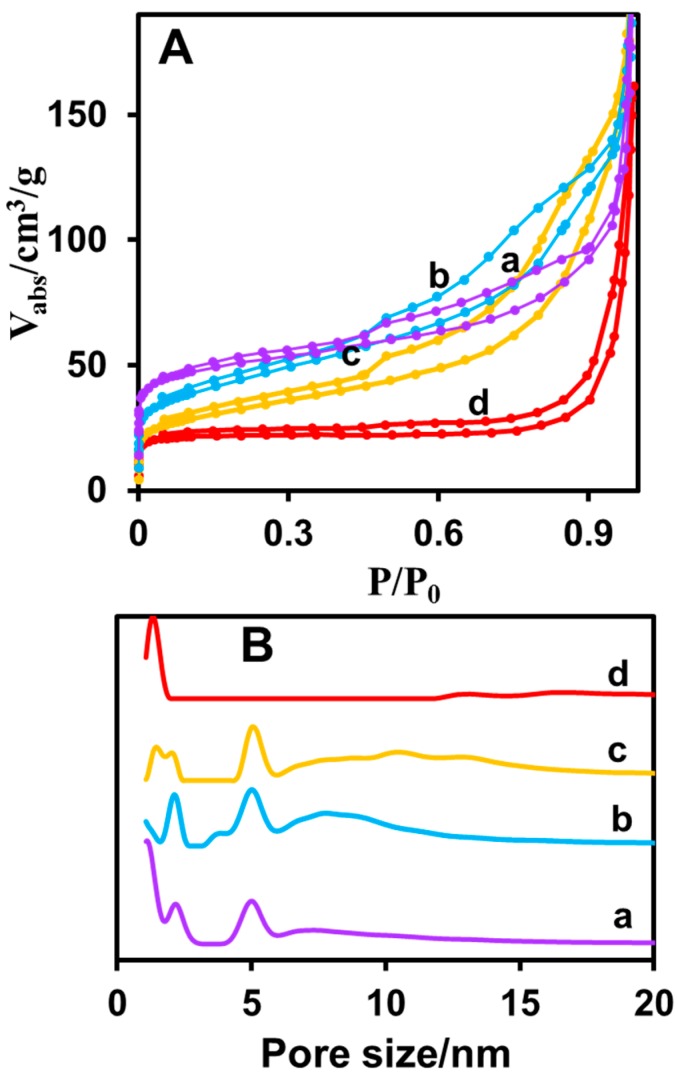
(**A**) N_2_ adsorption analysis of CuNPs/NGO (600 (a), 700 (b), 800 (c) and 900 °C (d)); (**B**) pore size distribution curves of CuNPs/NGO (600 (a), 700 (b), 800 (c) and 900 °C (d)).

The porosity and surface areas of the CuNPs/NGO materials were analyzed by N_2_ adsorption experiments at 77 K (Figure 4A). The results imply that the prepared samples possess a 3D network and the carbonization at high temperature under N_2_ atmosphere leads to nitrogen functionalized carbon content of the composites. The CMOFs are composed of multilayer amorphous carbon and additionally they have the essential influence of the accessibility of Glucose molecules into the CuNP catalytic surface sites. Even though the CMOFs have extremely decreased surface area, these materials unveiled interesting hierarchical porosity. Table 2 displays the surface area, porosity and pore volume of CuNPs/NGO materials. From Figure 4B, CuNPs/NGO (600 °C (a)), CuNPs/NGO (700 °C (b)), and CuNPs/NGO (800 °C (c)) displayed a considerable surface area and hierarchical porosity. The surface area (114 m^2^ g^−1^) of CuNPs/NGO ((800 °C) was slightly smaller compared to the surface areas of CuNPs/NGO (600 °C) and CuNPs/NGO (700 °C)). In addition, the existence of CuNPs into the composite at higher temperature changes the pore size distribution. In these materials, hierarchical porosity affords better accessibility of the glucose on catalytic sites of CuNPs/NGO nanocomposites, while CuNPs/NGO (900 °C (d)) material owns a small surface area and micro porous pore volume because of the presence of CuNPs on the surface of amorphous carbon layer. Thus, the better accessibility of glucose molecule is possible through the catalytic sites of CuNPs. As a result, a better non-enzymatic catalytic activity of the composite materials is expected at higher temperatures.

### 3.3. Electrochemical Behavior of CuNPs/NGO Nanocomposites

**Figure 5:**
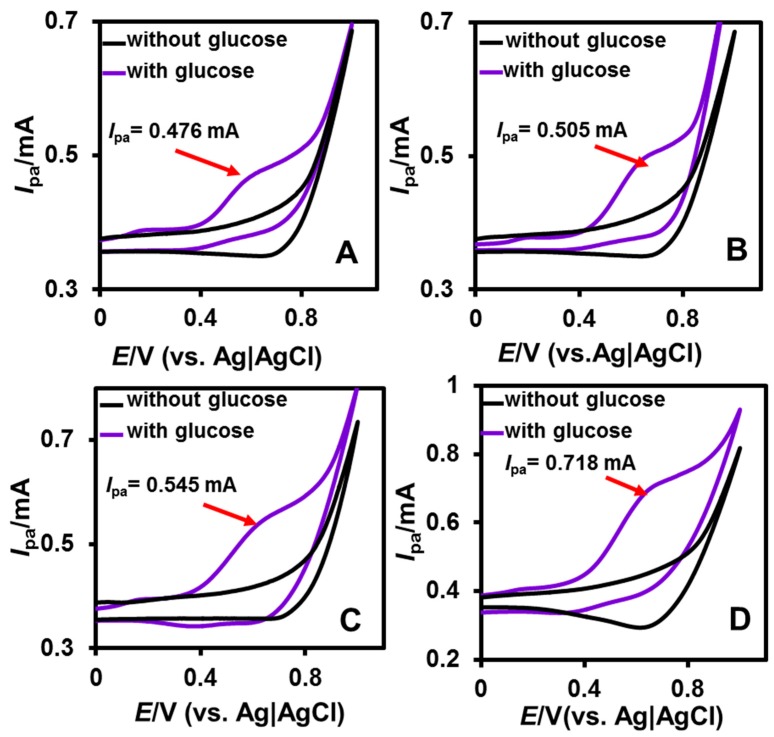
CVs obtained at CuNPs/NGO (600 (**A**), 700 (**B**), 800 (**C**) and 900 °C (**D**)) modified SPCEs in 0.1 NaOH with and without 3 mM glucose at the scan rate of 50 mV/s.

The electrochemical ability of unmodified and modified CuNPs/NGO (600, 700, 800 and 900 °C) screen printed electrode (SPCE) towards the oxidation of glucose was studied by the cyclic voltammetry (CV) method. The concentration of NaOH (0.1 M) and the scan rate (50 mV/s) were fixed for the cyclic voltammetric experiments. The unmodified SPCE showed the small anodic peak current towards the oxidation of glucose (Figure S4). This indicates the poor electron transfer, high over potential and electrode fouling of unmodified SPCE. However, a well-defined anodic peak appeared for the CuNPs/NGO (600 (A), 700 (B), 800 (C) and 900 °C (D) Figure 5) modified SPCEs in the presence of 1 mM glucose. A large anodic peak current (*I*_p_ = 0.706 mA) was observed for CuNPs/NGO (900 °C) towards the electro-oxidation of 1 mM glucose compared to other CuNPs/NGO (*I*_p_ = 0.476 mA (600 °C), *I*_p_ = 0.493 mA (700 °C) and *I*_p_ = 0.538 mA (800 °C)) modified electrodes. This can be due to the higher carbonization of CuNPs/NGO (900 °C) relative to others. Moreover, a large background current was also obtained for CuNPs/NGO (900 °C) compared to CuNPs/NGO (600, 700, and 800 °C) as presented in Figure 5. This observation revealed the synergistic effect between the good conductivity of NGO and good catalytic activity of CuNPs present in the prepared nanocomposites. To study the effect of loading at CuNPs/NGO (900 °C) towards electro-oxidation of glucose, we have chosen four different concentrations (0.5, 1, 2 and 2.5 mg/mL (DMF)) and their corresponding voltammograms were given in Figure S5. From the CVs, it revealed that maximum electrochemical ability was attained at 2 mg/mL loaded CuNPs/NGO (900 °C) modified SPCE. For the sake of clarity, CuNPs/NGO (900 °C) will be referred to as CuNPs/NGO for the remaining electrochemical studies. The possible electrochemical mechanism at CuNPs/NGO towards the oxidation of glucose is given below [39]:

(1) $$\mathrm{Cu}+{\mathrm{OH}}^{-}\to \mathrm{Cu}{\left(\mathrm{OH}\right)}_{2}+2e^{-},$$

(2) $$\mathrm{Cu}{\left(\mathrm{OH}\right)}_{2}+{\mathrm{OH}}^{-}\to \mathrm{CuOOH}+e^{-},$$

(3) $$\mathrm{CuOOH}+e^{-}+\mathrm{glucose}\to \mathrm{Cu}{\left(\mathrm{OH}\right)}_{2}+\mathrm{glucolactone}.$$

**Figure 6:**
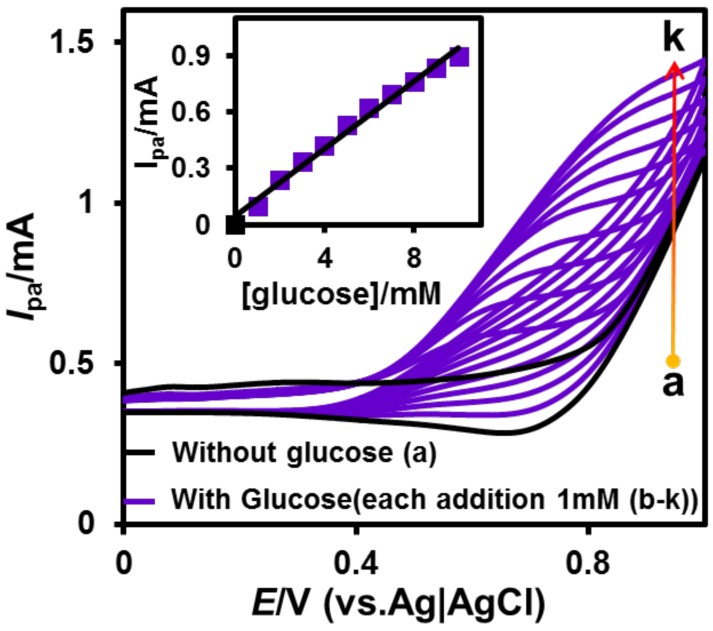
CVs observed without (**a**) and with each addition (**b**–**k**) of 1 mM glucose at CuNPs/NGO/SPCE in 0.1 M NaOH at the scan rate of 50 mV/s.

The cyclic voltammetry technique was also used to study the influence of glucose concentration at CuNPs/NGO modified SPCE. The experiment was carried out in 0.1 M NaOH at the fixed scan rate (50 mV/s) in the absence (a) and presence (b–k) of glucose (Figure 6). No noticeable anodic peak was observed at CuNPs/NGO/SPCE in the absence of glucose, whereas a well-defined and enhanced anodic peak appeared in the presence of 1 mM glucose. Moreover, a linear increase in the anodic peaks was attained upon increasing the concentration of glucose (each addition of 1 mM glucose). In addition, the linear dependency between *I*_p_ and (glucose) was observed in the equivalent calibration plot, indicating the good electrocatalytic ability of CuNPs/NGO/SPCE towards the determination of glucose.

### 3.4. Amperometric i–t Determination

**Figure 7:**
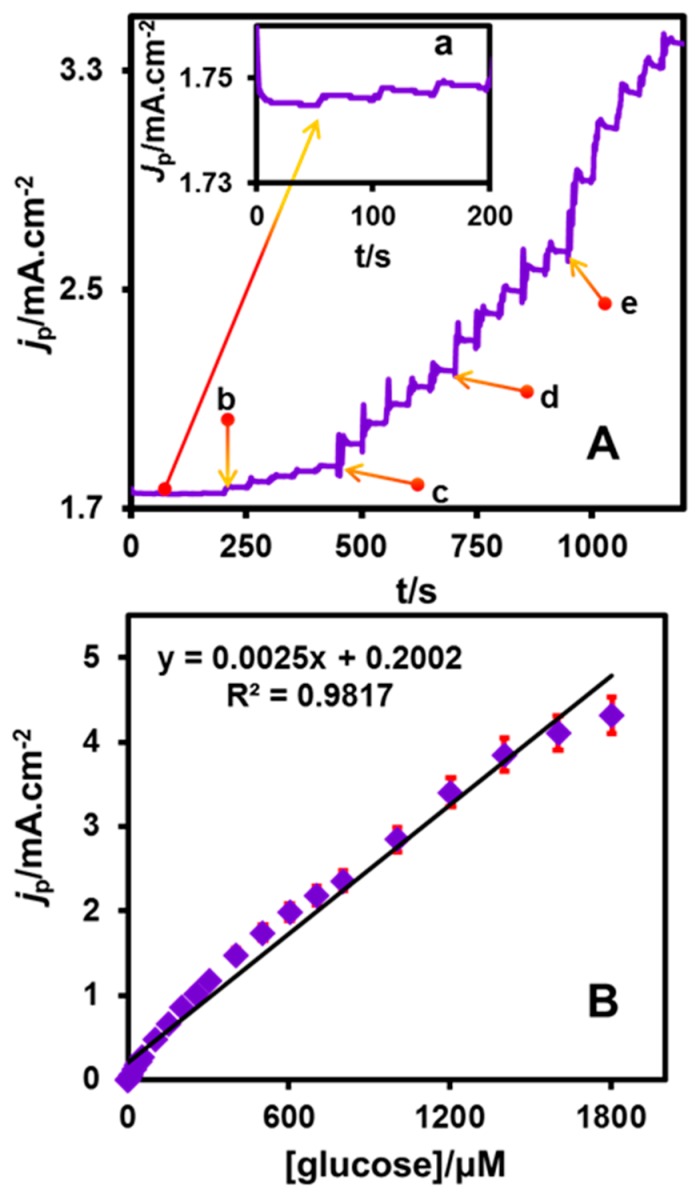
(**A**) amperometric response at CuNPs/NGO/SPCE upon successive additions of glucose (1, 10, 50, 100 and 200 μM (a–e)) of glucose in 0.1 M NaOH (scan rate = 50 mV s^−1^, applied potential = 0.4 V); (**B**) calibration plot of *I*_p_ vs. (*glucose*).

Figure 7A presents the amperometric i–t response at CuNPs/NGO/SPCE for the addition of varying glucose concentrations into the constantly stirred 0.1 M NaOH. The rotation speed and applied potential were held at 1500 rpm and 0.4 V. A quick and well-defined peak current response was observed for each consecutive addition of glucose (1, 10, 50, 100 and 200 μM (a–e)) indicating the outstanding catalytic ability of CuNPs/NGO modified SPCE towards the electro-oxidation of glucose. In addition, the 95.25% steady state current response attained within 3 s for each addition of glucose supports the excellent electrocatalytic activity of the fabricated electrode. The values of electroanalytical parameters such as linear range (1–1803 μM), limits of detection (LOD, 0.44 μM) and sensitivity (2500 μA mM^−1^ cm^−2^) were evaluated from the linear calibration plot between concentration of glucose and peak current response (Figure 7B). LOD = 3 *s*_b_/*S* (where, *s*_b_ = standard deviation of blank signal and *S* = sensitivity) was the equation applied in the calculation of sensitivity. The assessed electroanalytical parameters of CuNPs/NGO/SPCE were compared (Table 3) and found to be in close agreement with the earlier reported linear range, LOD and sensitivity values of the glucose sensors from the literature. The obtained sensitivity is higher, but the LOD is not so good compared with other examples [40,41,42,43,44,45,46,47,48,49]. This may be due to the size of the nanoparticles, and the amount of depositions of nanoparticles on the carbon materials.

### 3.5. Selectivity Studies

The experiment to examine the selectivity nature of CuNPs/NGO/SPCE towards glucose was performed in the presence of various interfering biomolecules, which can be oxidized at the same optimized potential (0.4 V) applied for the electro-oxidation of glucose. The selectivity of our modified electrode was studied (Figure S6) under the similar experimental conditions as mentioned in Section 3.4. Biological interferents such as sucrose, dopamine, uric acid, and ascorbic acid were taken in 0.1 mM concentration compared to glucose (1 mM). Since the concentration of chosen interferents is less than 0.1 mM in human blood serum, 0.1 mM concentration of common interferents have been chosen for the selectivity studies. An enhanced anodic peak current response was visualized at CuNPs/NGO modified SPCE with respect to 1 mM addition of glucose. In contrast, the addition of other interfering biomolecules (0.1 mM) showed no obvious peak response supporting the excellent catalytic activity of CuNPs/NGO/SPCE in the selective detection of glucose.

### 3.6. Repeatability, Reproducibility and Stability

The repeatability nature of CuNPs/NGO modified SPCE was investigated by executing six incessant experiments with single modified electrode towards 3 mM glucose at the scan rate of 50 mV/s. The corresponding relative standard deviation (RSD) value evaluated as 2.4% reveals the acceptable repeatability of our modified electrode. The reproducing capability was determined by conducting six independent measurements with six discreet fabricated electrodes. The obtained value of RSD (1.9%) exposes the appreciable reproducibility of CuNPs/NGO modified SPCE (Table S1). In order to test the stability, the anodic peak response of CuNPs/NGO/SPCE towards 3 mM glucose was recorded for a time period of 30 days, followed by storing the fabricated electrode at 4 °C in 0.1 M NaOH. During the daily experiments, a well-defined anodic peak current response was displayed by our modified electrode without any shift of peak potential towards 3 mM glucose. In addition, the final anodic peak response obtained at CuNPs/NGO modified SPCE was 96.2% of the initial peak current response (Table S2). Thus, our enzyme less glucose sensor holds considerable storage stability.

### 3.7. Real Sample Analysis

The demonstration of feasible practicality was done in the human serum samples collected from diabetes patients. The serum samples (2 mL) were diluted to 10 mL using 0.1 M NaOH and further utilized for the experiments under the above-mentioned experimental conditions in analyzing lab glucose samples. By employing the standard addition method, the concentration of glucose in the human serum samples were estimated as 5.16, 5.09, 6.73 and 6.49 mM. These values were in accordance with the values of glucose concentration (5.31, 5.05, 6.85 and 6.61 mM) evaluated by using the commercially available Tecan Sunrise plate reader (Table S3). The adequate recovery results imply the successful practicability of our CuNPs/NGO modified SPCE.

## 4. Conclusions

A highly selective amperometric glucose sensor was constructed based on the as-prepared CuNPs/NGO, working in a wide linear range from 1 to 1803 μM. Our modified constructed electrode holds low limits of detection (0.44 μM) and high sensitivity (2500 μA mM^−1^ cm^−2^). The unusually precise detection of glucose in the human serum samples exposes the fabricated electrode as a peculiar real-time glucose sensor. The additional advantages such as facile operational approach, increased porosity and outstanding catalytic activity towards the determination of glucose prolong the usage of CuNPs/NGO/SPCE for batteries, biosensors and super capacitors in the immediate future.

## Figures and Tables

**Table 1:** Atom distribution in the Cu nanoparticles decorated nitrogen doped graphite oxide, which was assessed by using ICP-MS (Cu) and elemental analysis (N, C, H and O).

Sample	Cu wt %	N wt %	C wt %	H wt %	O wt %
CuNPs/NGO(600)	71.63	2.10	22.84	1.72	1.71
CuNPs/NGO(700)	71.68	1.96	22.73	1.79	1.84
CuNPs/NGO(800)	71.34	1.94	23.82	1.83	1.07
CuNPs/NGO(900)	77.43	1.44	17.21	1.19	2.73

**Table 2:** The BET surface areas and pore sizes of CuNPs/NGO (600, 700, 800 and 900).

Sample	Surface Area/m^2^ g^−1^		Total Pore Volume (cc/g) *P*/*Po* ~ 0.99	Pore Size (nm)
	BET	Langmuir		
CuNPs/NGO(600)	185	193	0.31	2, 5
CuNPs/NGO(700)	144	151	0.34	2, 3–5
CuNPs/NGO(800)	108	113	0.32	1–2, 5
CuNPs/NGO(900)	86	90	0.25	1.2

**Table 3:** Comparison of electroanalytical parameters for the determination of glucose at CuNPs/NGO nanocomposite modified electrode with previously reported modified electrodes.

Modified Electrode	^a^ LR (μM)	^b^ LOD (μM)	Sensitivity	Ref.
3D N-Co-CNT@NG)	25–10,830	0.1	9.05 µA mM^−1^ cm^−2^	[40]
Cu@porous carbon	1–6000	0.6	10,100	[41]
[Cu_3_(^c^ btc)_2_] nanocube	1–2250	1	549 µA mM^−1^ cm^−2^	[42]
Cu-^d^ BDD	1–50	10	2.3 µA mM^−1^ cm^−2^	[43]
N-doped Carbon-Cu nanohybrids	5–2100	0.7	223.6 µA mM^−1^ cm^−2^	[44]
Graphene oxide and NiO nanofibers	2–600	0.77	1100	[45]
Cu nanoporous	10–500	40	220 µA mM^−1^ cm^−2^	[46]
Cu NPs/SWCNTs	0.5–500	0.3	0.256 µA mM^−1^ cm^−2^	[47]
Cu/CuO/ZnO	100–1000	18	408 µA mM^−1^ cm^−2^	[48]
AuCu/CNTs	80–9260	4	22 µA mM^−1^ cm^−2^	[49]
CuNPs/NGO	1–1803	0.44	2500 µA mM^−1^ cm^−2^	This work

^a^ LR—linear range, ^b^ LOD—limits of detection, ^c^ btc—Benzene-1,3,5-tricarboxylate, ^d^ BDD—boron doped diamond electrode.

## Supplementary Materials

The following are available online at http://www.mdpi.com/2079-4991/8/6/429/s1. Figure S1. PXRD pattern of as-synthesized MOF [Cu_2_(BDC)_2_(DABCO)]. Figure S2. N_2_ adsorption analysis of activated MOF [Cu_2_(BDC)_2_(DABCO)]. Figure S3. Raman spectra fitted with Gaussian profiles of CuNPs/NGO (carbonized at 600 °C (a), 700 °C (b), 800 °C (c) and 900 °C (d)). Figure S4 CVs without (a) and with each addition (b–k) of 3 mM glucose at unmodified SPCE in 0.1 M NaOH at the scan rate of 50 mV/s. Figure S5. Voltammograms obtained at CuNPs/NGO modified SPCE for different loading concentrations (0.5, 1, 2 and 2.5 mg/mL (DMF)) of CuNPs/NGO. Figure S6. Selectivity study of CuNPs/NGO modified SPCE towards glucose (a) in presence of sucrose (b), dopamine (c), uric acid (d) and ascorbic acid (e). Table S1: Repeatability study at CuNPs/NGO modified SPCE in 5 different solutions towards 3 mM Glucose and Reproducibility study at 5 different CuNPs/NGO modified SPCEs towards 3 mM Glucose. Table S2: Stability study of 3 mM Glucose at CuNPs/NGO modified SPCE. Table S3. Determination of glucose in human blood serum samples.
