# Supplementary material for: Copper Nanoparticle and Nitrogen Doped Graphite Oxide Based Biosensor for the Sensitive Determination of Glucose
Source: Nanomaterials (Basel). 2018 Jun 13;8(6):429. doi: 10.3390/nano8060429 (PMC6027177; doi:10.3390/nano8060429)
Supplement: Supplementary file 1 [file nanomaterials-08-00429-s001.zip › nanomaterials-312027-SI.pdf]

Supporting Information

Copper Nanoparticle and Nitrogen Doped Graphite Oxide Based Biosensor for the Sensitive Determination of Glucose

Kulandaivel Sivasankar[a], Karuppasamy Kohilarani[b], Sea-Fue Wang[*b], Rajkumar Devasenathipathy[b], Chia-Her Lin[*a,c]

[a]Department of Chemistry, Chung-Yuan Christian University, Chungli, Taiwan 32023. E-mail: chiaher@cycu.edu.tw; Fax: +886-3-2653399; Tel: +886-3-2653315.

[b]Department of Materials and Mineral Resources Engineering, No. 1, Sec. 3, Chung-Hsiao East Rd., National Taipei University of Technology, Taipei, Taiwan E-mail:sfwang@ntut.edu.tw

[c]R&D Center for Membrane Technology, Chung Yuan Christian University.

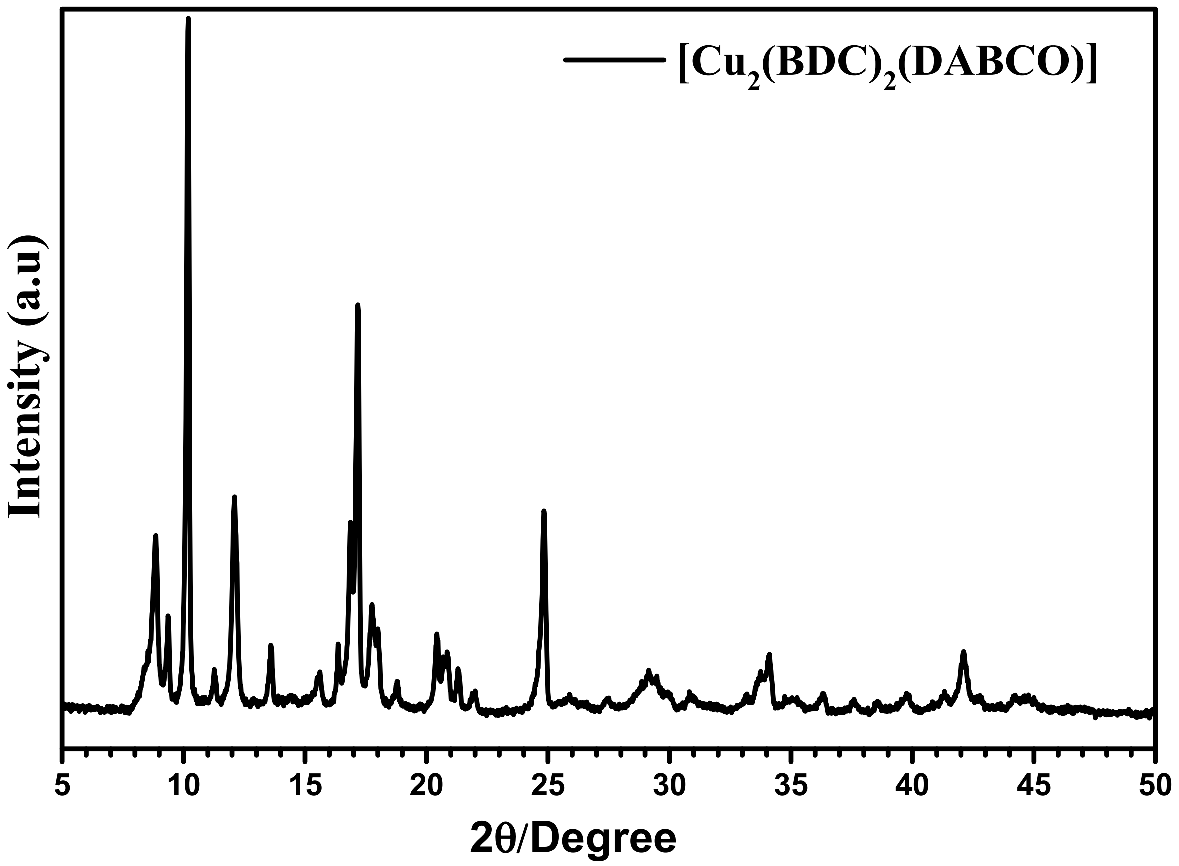

Figure S1. PXRD pattern of as-synthesized MOF [$Cu_2(BDC)_2(DABCO)$].

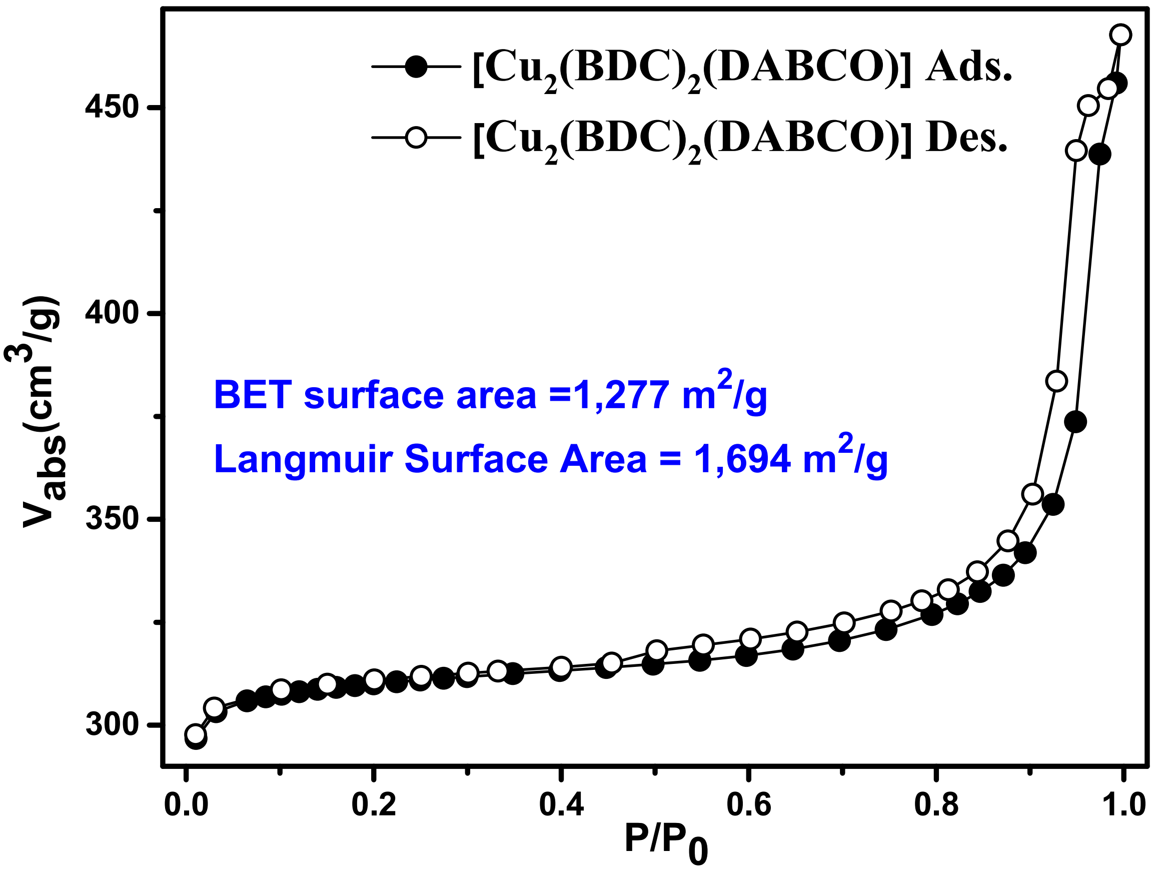

Figure S2. N_2 adsorption analysis of activated MOF [$Cu_2(BDC)_2(DABCO)$].

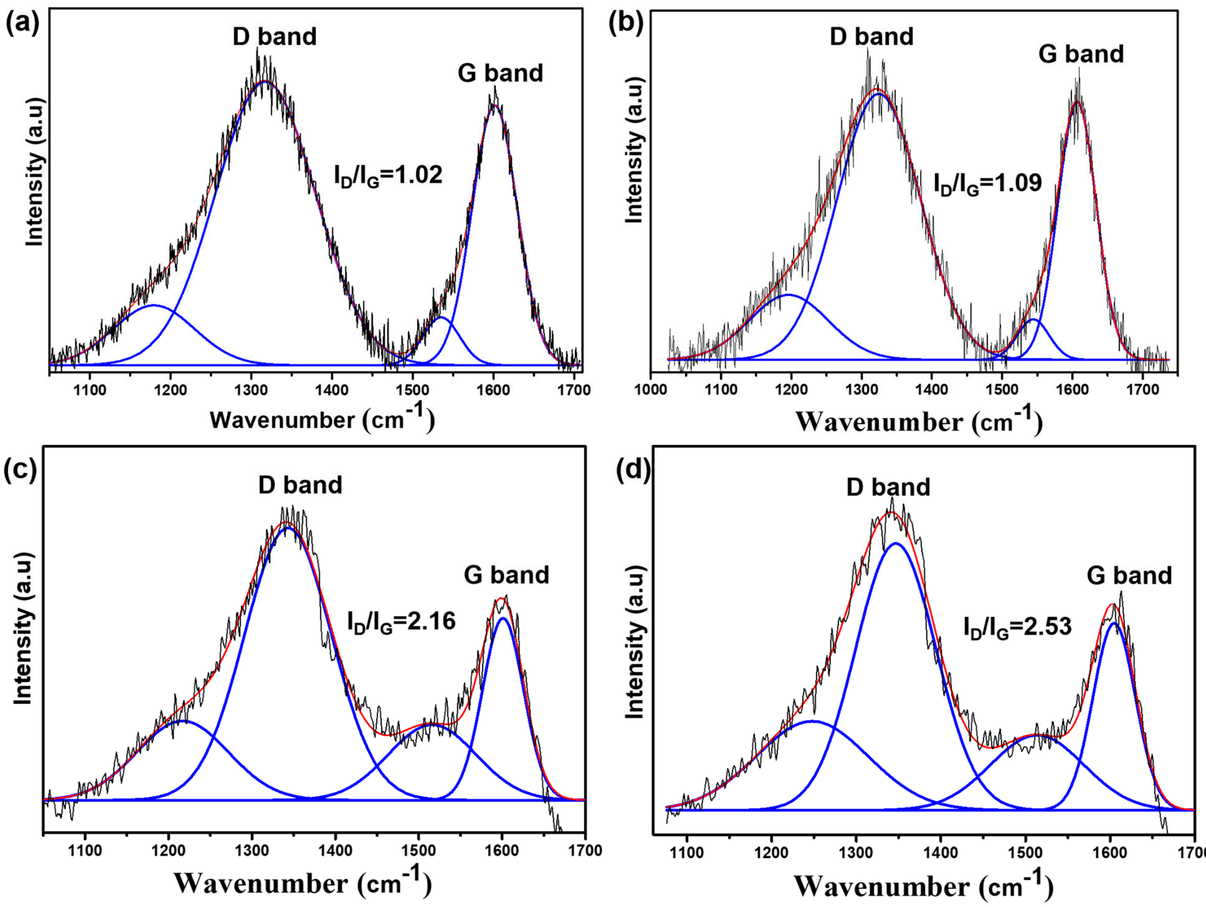

Figure S3. Raman spectra fitted with Gaussian profiles of CuNPs/NGO (carbonized at 600 °C (a), 700 °C (b), 800 °C (c) and 900 °C (d))

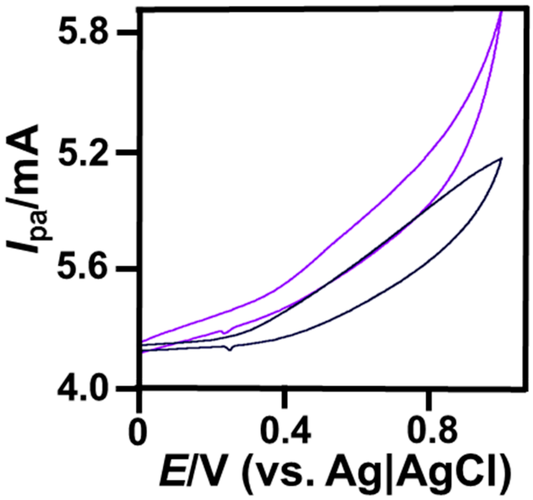

Figure S4 CVs without (a) and with each addition (b-k) of 3 mM glucose at unmodified SPCE in 0.1 M NaOH at the scan rate of 50 mV/s

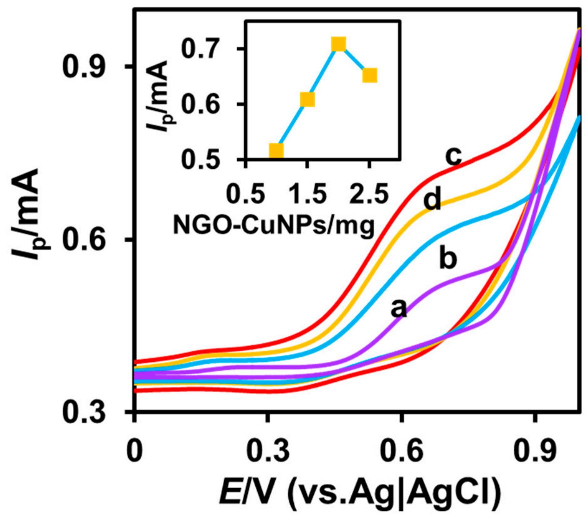

Figure S5. Voltammograms obtained at CuNPs/NGO modified SPCE for different loading concentrations (0.5, 1, 2 and 2.5 mg/ml (DMF)) of CuNPs/NGO

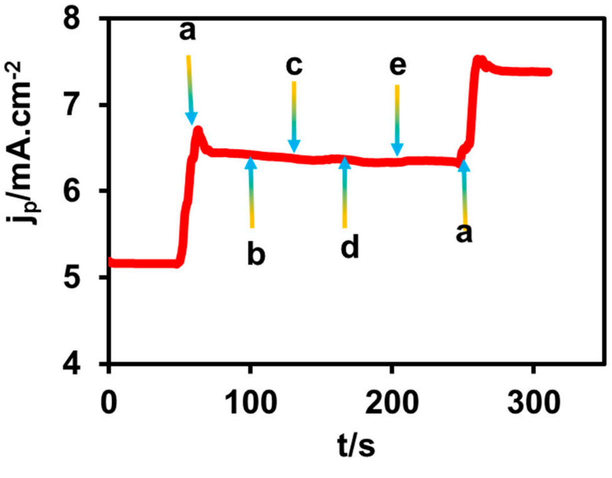

Figure S6. Selectivity study of CuNPs/NGO modified SPCE towards glucose (a) in presence of sucrose (b), dopamine (c),uric acid (d) and ascorbic acid (e).

Table S1. Repeatability study at CuNPs/NGO modified SPCE in 5 different solutions towards 3 mM Glucose and Reproducibility study at 5 different CuNPs/NGO modified SPCEs towards 3 mM Glucose

Repeatability		Reproducibility	
Solution	Current (mA)	Electrode	Current (mA)
1	0.701	1	0.714
2	0.688	2	0.721
3	0.721	3	0.713
4	0.713	4	0.696
5	0.683	5	0.692
6	0.724	6	0.726
Average	0.705	Average	0.710
SD	0.01715	SD	0.01355
RSD	2.43	RSD	1.91

Table S2. Stability study of 3 mM Glucose at CuNPs/NGO modified SPCE

Time (Day)	Current (mA)	Current Retained (%)
0	0.723	100
5	0.718	99.3
10	0.706	97.6
20	0.701	96.9
30	0.696	96.2

Table S3. Determination of glucose in human blood serum samples.

Serum Sample	Glucose detected by commercial detector(mM)	Glucose detected by our sensor	Recovery (%)	RSD
1	5.31	5.16	97.15	2.3
2	5.05	5.09	100.7	1.93
3	6.85	6.73	98.24	2.42
4	6.61	6.49	98.18	2.19

RSD – Relative standard deviation for three measurements.
